# Effect of Methylmercury Exposure on Bioaccumulation and Nonspecific Immune Respsonses in Hybrid Grouper *Epinephelus fuscoguttatus* × *Epinephelus lanceolatus*

**DOI:** 10.3390/ani12020147

**Published:** 2022-01-08

**Authors:** Hsiang-Chieh Chuang, Huai-Ting Huang, Novi-Rosmala Dewi, Hsi-Hua Hsiao, Bo-Ying Chen, Zhen-Hao Liao, Meng-Chou Lee, Po-Tsang Lee, Yu-Sheng Wu, Yu-Ju Lin, Fan-Hua Nan

**Affiliations:** 1 Department and Graduate Institute of Aquaculture, National Kaohsiung University of Science and Technology, Kaohsiung City 811213, Taiwan; hcchuang@nkust.edu.tw; 2 Department of Aquaculture, National Taiwan Ocean University, Keelung City 20224, Taiwan; twinkleqazwsx784@gmail.com (H.-T.H.); novi.rosmala.d@gmail.com (N.-R.D.); 10933032@mail.ntou.edu.tw (H.-H.H.); joey860812@gmail.com (B.-Y.C.); smallhowhow1234@gmail.com (Z.-H.L.); mengchoulee@mail.ntou.edu.tw (M.-C.L.); leepotsang@mail.ntou.edu.tw (P.-T.L.); 3 Department of Aquaculture, National Pingtung University of Science and Technology, Pingtung County 912301, Taiwan; wuys0313@mail.npust.edu.tw; 4 Department of Life Sciences, National Chung Hsing University, Taichung City 402002, Taiwan; yjl@dragon.nchu.edu.tw

**Keywords:** hybrid grouper, methylmercury, bioaccumulation, immunity

## Abstract

**Simple Summary:**

The head kidney was primary organ that accumulated methylmercury in hybrid grouper. Muscle tissue had lower methylmercury content than the head kidney and liver. Nonspecific immune responses and bioaccumulation of methylmercury were linked to hybrid grouper health.

**Abstract:**

Mercury (Hg) is a dangerous heavy metal that can accumulate in fish and is harmful when consumed by humans. This study investigated the bioaccumulation of mercury in the form of methylmercury (MeHg) and evaluated nonspecific immune responses such as phagocytic activity and superoxide anion (O_2_^−^) production in hybrid grouper (*Epinephelus fuscoguttatus* × *E. lanceolatus*). The hybrid grouper leukocytes were incubated with methylmercury chloride (CH_3_HgCl) at concentrations of 10–10,000 µg/L to determine cell viability, phagocytic activity, and O_2_^−^ production in vitro. Subsequently, the grouper were exposed daily to CH_3_HgCl mixed in the experimental diets at concentrations of 0, 1, 5, and 10 mg/kg for 28 days. The bioaccumulation of MeHg in the liver, head kidney, and muscle tissue was measured, and the phagocytic activity and O_2_^−^ production were evaluated. In vitro results indicated that cell viability was significantly lower than that of the control group at concentrations > 500 µg/L. The phagocytic rate and O_2_^−^ production at concentrations ˃ 500 and ˃ 200 µg/L, respectively, were significantly lower than those of the control group. The dietary exposure demonstrated that MeHg accumulated more substantially in the liver and head kidney compared with the muscle tissue in the treatment groups. Moreover, the cumulative concentration significantly increased with higher concentrations and more days of exposure. The phagocytic rate and O_2_^−^ production in the treatment groups were significantly lower than those in the control group from days 2 and 1, respectively. In conclusion, hybrid grouper accumulated significant MeHg in the liver and head kidney compared with the muscle tissue, and higher concentrations and more exposure days resulted in decreased cell viability, phagocytic activity, and O_2_^−^ production.

## 1. Introduction

Grouper is an important aquaculture species cultivated in Taiwan. As the world’s second largest grouper producer, Taiwan’s production of approximately 234 tons accounted for 10% of the world’s total grouper production in 2019 [1]. Cultured species are primarily tiger grouper (*Epinephelus fuscoguttatus*) and giant grouper (*E. lanceolatus*), which are widely distributed in the Indo-Pacific region [2]. Tiger grouper is a popular cultured marine species in Asia due to its rapid growth rate, and giant grouper, which can reach a maximum weight of 400 kg, is the largest grouper species [3]. A crossbreed of these two species is the hybrid giant tiger grouper, which became popular due to its high survival rate, feeding performance, rapid growth rate, and tolerance of a wide range of rearing parameters [4,5]. Grouper aquaculture is mostly conducted in offshore cages; marine culture, which first developed in the 1990s for cobia (*Rachycentron canadum*), is a current trend in raising giant grouper [6]. However, marine environmental pollution from heavy metals has attracted global attention because such pollution harms fish [7].

Heavy metals such as cadmium (Cd), mercury (Hg), lead (Pb), chromium (Cr), and arsenic (As) are toxic to both humans and animals [8]. Mercury is a toxic heavy metal and has no essential biological function. Commonly, mercury enters the marine environment through natural processes such as volcanic activity and erosion or industrial and agricultural sewage discharge; mercury affects the food chain through fish consumption and can affect human health [9]. Even MeHg is present in very low concentrations in seawater, they are absorbed by algae at the start of the food chain. The algae is then eaten by fish and other higher-level organisms in the food chain. Methylmercury (MeHg) and inorganic Hg(II) are the most abundant forms of Hg found in fish [10]. Due to industrial activities, aquatic animals absorb more and more pollutants, including MeHg. Fish can efficiently absorb MeHg but excrete very slowly. Several studies have focused on the human health risk associated with exposure to toxic metals through fish consumption [10,11,12]. However, how bioaccumulation and nonspecific immune response affect fish health has been neglected. Few studies have addressed the fish health risk associated with MeHg toxicity, specifically in terms of physiological responses [13,14].

Fish meal which is made from whole fish or fish related product is one of the common feed ingredients. Fish meal that made from these contaminated fish contains highly MeHg. Grouper culture may be affected by MeHg pollution of its environment because fish meal-containing feed is used in the culture system. However, little is known regarding the risk of such pollution to the hybrid grouper. To our knowledge, no studies have focused on the effect of MeHg toxicity, nonspecific immune responses, and bioaccumulation on the hybrid grouper. The head kidney is a lymphatic organ and produce a lot of leukocytes which are involved in phagocytosis of invading pathogens [15]. Head kidney play a key role in the nonspecific immune response of fish. Fish consumption is the main source of exposure to methylmercury for human, and grouper is an important economic aquatic species in Taiwan. Therefore, it is very important to understand the safety of grouper in food and breeding. In our study, the effect of MeHg on phagocytic activity and O_2_^−^ production of grouper head kidney leukocytes were evaluated in vitro and in vivo. The bioaccumulation of MeHg in the liver, head kidney, and muscle tissue was measured to determine the accumulation level after dietary exposure.

## 2. Materials and Methods

### 2.1. The In Vitro Effect of Methylmercury

Cell viability and nonspecific immune responses in vitro were evaluated to determine the suitable concentration to use in in vivo experiment. Each in vitro experiment was performed with 3 hybrid groupers (200 ± 24.98 g). Hybrid groupers were purchased from local farm in Yilan County, Taiwan. The fish were acclimatized to the laboratory conditions for 2 weeks in tanks (200 × 120 × 70 cm) and fed with a commercial diet (Tairoun Products Co., Ltd., Taiwan) twice daily. The feed contains 45% crude protein, 8% crude lipid, 16% ash, and 3% crude fibre. All the fish appeared with normal activity, feed intake and without injury. The water conditions were maintained as follows: temperature at 27 ± 1 °C, salinity at 33 ± 1‰, the pH at 8.2 ± 0.2, dissolved oxygen (DO) at 5.5–7.3 mg/L, and a photoperiod of 12 h of light: 12 h of dark. Animal experiments were performed according to the guidelines of the Institutional Animal Care and Use Committee at National Taiwan Ocean University (protocol number 105063).

The head kidneys of fish were excised, minced and mixed together. Leukocytes were isolated from the head kidney of the fish using the Percoll density gradient centrifugation method modified from Chang et al. [16]. Two Percoll mixtures were prepared from Percoll (Pharmacia, Piscataway, NJ, USA) and NaCl (1.5 M, Sigma-Aldrich, St. Louis, MO, USA) at a ratio of 9:1 and HBSS (Hank’s balanced salt solution; Gibco, Waltham, MA, USA) at concentrations of 35% and 50%. The head kidney was placed in a petri dish containing 4 mL of HBSS. Briefly, the head kidney was shredded with tweezers and filtered through a 100-µm nylon mesh (Bio-Rad, Hercules, CA, USA). The filtrate was put into a centrifuge tube filled with 35% and 50% Percoll mixtures and centrifuged at 400× *g*, 4 °C, for 10 min. The leukocytes at the interface were collected and subsequently resuspended with HBSS and centrifuged; this procedure was repeated three times. The leukocyte concentration was adjusted to 5 × 10^6^ cells/mL.

#### 2.1.1. Cell Viability

Cell viability was evaluated using an MTT assay by following the method of Domínguez-Borbor et al. [17] with modifications. Briefly, 100 µL of a leukocyte suspension was pipetted into each plate of a 96-well microplate, which was then centrifuged at 400× *g*, 4 °C, for 10 min. The supernatant was removed, and 100 µL of CH_3_HgCl in HBSS at concentrations of 0, 10, 50, 70, 100, 200, 500, 1000, 5000, and 10,000 µg/L was added into each well in triplicate. The cells were incubated for 30 min. Subsequently, the supernatant was removed, and 100 µL of MTT solution was added into each well in the dark and incubated for 4 h at room temperature. After removing the supernatant, 100 µL of dimethyl sulfoxide (DMSO; Honeywell, Charlotte, NC, USA) was added and the resultant mixture was vortexed for 10 min. Absorbance was measured using an ELISA reader (VersaMax Tunable Microplate Reader; Molecular Devices, Sunnyvale, CA, USA) at a wavelength of 570 nm. The cell viability percentage was calculated using the following equation:

(1) $$\mathrm{Cell}\;\mathrm{viability}\;(\%)=\frac{OD\;of\;treatment\;average}{OD\;of\;control\;average}\times 100\%$$

#### 2.1.2. Phagocytic Activity Assay

The phagocytic activity in vitro was evaluated after preincubating with CH_3_HgCl at concentrations of 0, 50, 100, 200, 500, 1000, and 5000 µg/L for 30 min. Phagocytic activity was evaluated using a method modified from Yue et al. [18]. Briefly, 100 µL of leukocyte suspension was dropped on the cover glass and incubated for 1 h at room temperature. The supernatant was removed, and 100 µL of latex beads suspension (0.8 µm, 5 × 10^7^ beads/mL; Sigma-Aldrich, St. Louis, MO, USA) was added and incubated for 30 min at room temperature. The non-phagocytosed beads were removed with HBSS and fixated with methanol for 5 min. Subsequently, the surface was washed with ddH_2_O, stained with Giemsa for 20 min, decolorized with ddH_2_O, air-dried, and observed under a light microscope (Olympus BX41 Phase Contrast; Olympus, Tokyo, Japan). The phagocytic cells were counted, and the phagocytic rate and phagocytic index were calculated using the following equations:

(2) $$\mathrm{Phagocytic}\;\mathrm{rate}\;(\%)=\frac{Total\;phagocytic\;cells}{Total\;cells}\times 100$$

(3) $$\mathrm{Phagocytic}\;\mathrm{index}=\frac{Beads\;in\;phagocytic\;cells}{Total\;phagocytic\;cells}$$

#### 2.1.3. Superoxide Anion (O_2_^−^) Production Assay

The O_2_^−^ production rate was evaluated using the nitro blue tetrazolium reduction method modified from Rotllant et al. [19]. Briefly, 100 µL of a leukocyte suspension was pipetted into each plate of a 96-well microplate, which was then centrifuged at 400× *g*, 4 °C, for 10 min. The supernatant was removed, and 100 µL of CH_3_HgCl in HBSS at concentrations of 0, 50, 100, 200, 500, 1000, and 5000 µg/L was added into each well in triplicate (For in vivo experminent, the step of incubation was skipped). After 30 min incubation, the supernatant was removed, and 100 µL of zymosan (Sigma-Aldrich, St. Louis, MO, USA) for the sample and 100 µL of HBSS for the background were added; the solution was incubated for 30 min at room temperature. The supernatant was removed, 100 µL of 0.3% NBT solution was added, and the solution was incubated for 30 min at room temperature. The supernatant was removed and washed with methanol; this procedure was repeated three times. After the supernatant was air-dried, 120 µL of KOH (2M) and 140 µL of DMSO were added, and the absorbance was measured using an ELISA reader at a wavelength of 630 nm. The O_2_^−^ production rate was calculated using the following equation:

(4) $${O_{2}}^{-}\;\mathrm{production}\;\mathrm{rate}\;(\%)=\frac{OD\;of\;sample\;–\;OD\;of\;background}{OD\;of\;background}\times 100$$

### 2.2. The In Vivo Effect of Methylmercury

#### 2.2.1. Dietary Exposure of Methylmercury

For experimental diets, the commercial feed was crushed and divided into four groups. Group 1 (control group) was not mixed with others. Group 2–4 were mixed with 1, 5 and 10 mg methylmercury chloride standard solution (CH_3_HgCl; purity 99.6%; Sigma-Aldrich, St. Louis, MO, USA) per kg. Subsequently, the feed were pelleted through a miner; the resulting pellets were dried in an oven at 40 °C and stored in a dark sealed bin. The concentration of MeHg in each feed was confirmed using high performance liquid chromatography–inductively coupled plasma–mass spectrometry (HPLC-ICP-MS).

A total of 87 hybrid groupers (200 ± 24.98 g) were randomly distributed into four groups. Each group included 21 fish maintained in a 270 L tank (90 × 60 × 50 cm) with flow through system, and water exchange was 50% per day. The fish was fed twice daily with the experimental diets at 3% body mass for 28 days. Three fish from each group were sampled on days 0, 1, 2, 7, 14, and 28 to determine immune parameters of head kidney leukocytes and bioaccumulation of MeHg in liver, head kidney, and muscle. The phagocytic activity and O_2_^−^ production were measured following the methods described in Section 2.1.2 and Section 2.1.3 using leukocytes isolated from the head kidney. The samples of bioaccumulation were from the same fish which from the nonspecific immune samples.

#### 2.2.2. Measurement of Methylmercury Bioaccumulation

Bioaccumulation of MeHg in the liver, head kidney, and muscle tissue was determined using the high performance liquid chromatography -inductively coupled plasmamass spectrometry (HPLC-ICP-MS) method. Each group has three replications. Briefly, the sample was minced with a homogenizer, and 0.5 g of the sample was put in a flask, mixed with extraction solvent (10 g of L-cysteine hydrochloride in 1000 mL of deionized water), and extracted by shaking vigorously for 10 s, followed by ultrasonic shaking in a 60 °C water bath for 40 min. The volume was adjusted to 50 mL at room temperature and filtered through a 0.2-µm filter membrane (Biofil, Indore, MP, India). Extraction solvent (treated equally) was used as a blank solution.

HPLC-ICP-MS was performed using an inductively coupled plasma mass spectrometer (Thermo X series II; Thermo Fisher Scientific, Waltham, MA, USA) equipped with a P680 HPLC pump (Dionex, Sunnyvale, CA, USA). HPLC separation was performed in a Synergi Hydro-RP 80 Å C18 column (150 × 4.6 mm i.d., 4.0-µm particle size; Phenomenex, Torrance, CA, USA) with a mobile phase of L-cysteine and L-cysteine hydrochloride (1 g each, dissolved in 1000 mL of deionized water) at a flow rate of 1.0 mL/min at 25 °C. The concentration of MeHg in each sample was confirmed using HPLC-ICP-MS and eluted with the mobile phase for 5 min. Standard solutions over a range of MeHg concentrations (0.4–10 µg/L) spiked with Rhodium (10 ppb) was used to generate a calibration curve (*r*^2^ = 0.9999). The analytical procedure was validated by a matrix spike and MeHg recovery rate was found to be more than 95%. The reproducibility CV (coefficient of variation) was 2.07%. The concentration of MeHg in the samples was calculated using the following equation:

(5) $$\mathrm{MeHg}\;(\mathrm{ppm})=\frac{\left(C-C_{0}\right)\times V}{W\times 1000}$$

*C*: Concentration of methylmercury in the test solution (ng mL^−1^)

*C_0_*: Concentration of MeHg in the blank solution (ng mL^−1^)

*V*: Final volume of the sample (mL)

*W*: Weight of the sample (g)

### 2.3. Statistical Analysis

Each experiment was conducted in triplicates and data was shown as mean±SD (standard deviation). One-way ANOVA and Tukey’ test were performed to compare the differences between groups at indicated time point or between different time points in a group Statistical Analysis Software version 9.4 (SAS, Cary, NC, USA). Two-way multivariate analysis of variance (MANOVA) was used to analyse the effects of concentrations and days of exposure. Significant differences among data were further analyzed using Tukey’s honestly significant differences procedure with *p* values < 0.05 considered significant.

## 3. Results

### 3.1. Effect of Methylmercury on Cell Viability In Vitro

**Figure 1:**
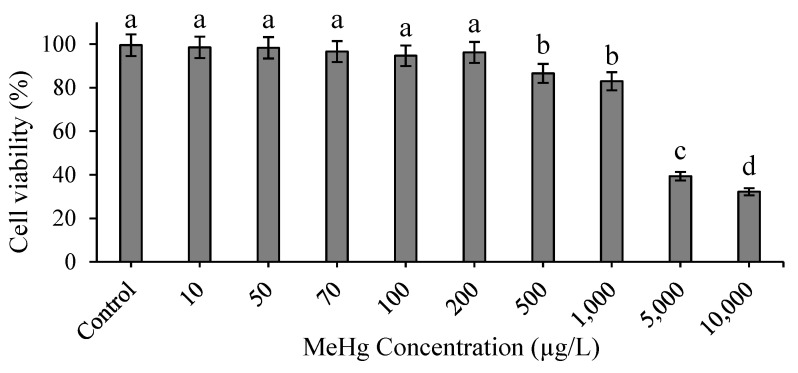
Cell viability of hybrid grouper leukocytes after in vitro incubation with various concentrations of methylmercury for 30 min. One-way ANOVA and Tukey’ test were performed to compare the differences between groups. Values are presented as mean ± SD (*n* = 3). Significant differences (*p* < 0.05) between treatment groups are indicated by different letters above the bars.

The cell viability values at concentrations of 10, 50, 70, 100, and 200 µg/L were ˃ 95% and had no significant differences compared with the control group (Figure 1). Conversely, the cell viability values at concentrations of 500, 1000, 5000, and 10,000 µg/L decreased by 86%, 82%, 39%, and 32%, respectively, and had significant differences compared with the control group (*p* < 0.05).

### 3.2. Effect of Methylmercury on Nonspecific Immune Responses In Vitro

**Figure 2:**
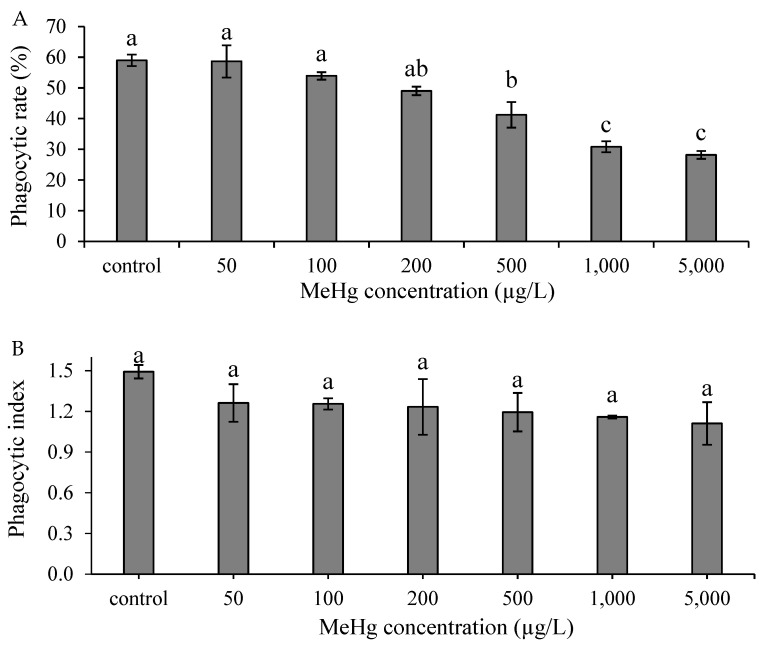
Phagocytic rate (**A**) and phagocytic index (**B**) of hybrid grouper leukocytes after in vitro incubation with various concentrations of methylmercury for 30 min. One-way ANOVA and Tukey’ test were performed to compare the differences between groups. Values are presented as mean ± SD (*n* = 3). Significant differences (*p* < 0.05) between treatment groups are indicated by different letters above the bars.

**Figure 3:**
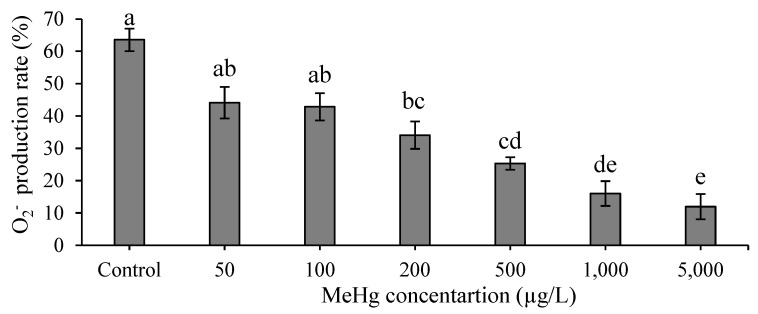
Superoxide anion production rate of hybrid grouper leukocytes after in vitro incubation with various concentrations of methylmercury for 30 min. One-way ANOVA and Tukey’ test were performed to compare the differences between groups. Values are presented as mean ± SD (*n* = 3). Significant differences (*p* < 0.05) among treatment groups are indicated by different letters above the bars.

The phagocytic rate indicated no significant differences at concentrations of 50, 100, or 200 µg/L, whereas at concentrations of 500, 1000, and 5000 µg/L, the rate indicated significant differences compared with the control group (*p* < 0.05; Figure 2A). Moreover, the phagocytic index indicated no significant difference at all concentrations compared with the control group (Figure 2B). Additionally, the O_2_^−^ production rate had no significant differences at concentrations of 50 and 100 µg/L compared with the control group (*p* > 0.05; Figure 3). There are significant differences at concentrations of 200, 500, 1000, and 5000 µg/L compared with the control group (*p* < 0.05; Figure 3).

### 3.3. Effect of Methylmercury Dietary Exposure on Nonspecific Immune Responses

**Figure 4:**
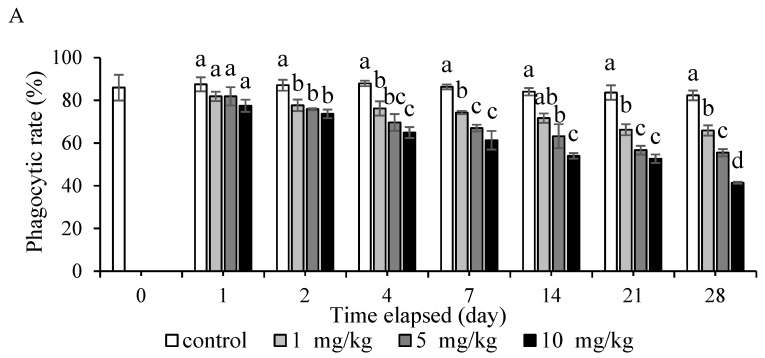

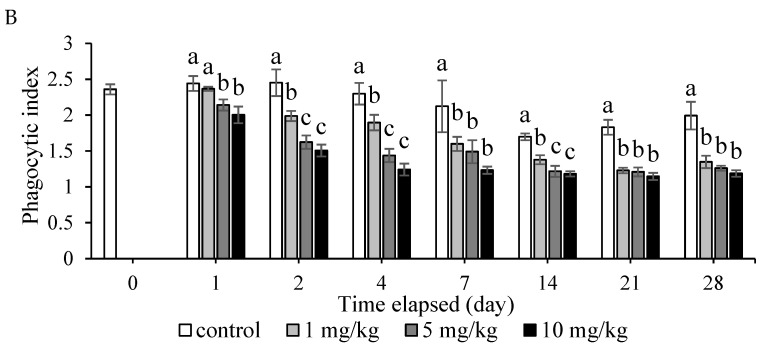
Phagocytic rate (**A**) and phagocytic index (**B**) of hybrid grouper fed with various concentrations of methylmercury for 28 days. One-way ANOVA and Tukey’ test were performed to compare the differences between groups at indicated time point. Values are presented as mean ± SD (*n* = 3). Significant differences (*p* < 0.05) between treatment groups are indicated by different letters above the bars.

Hybrid groupers were exposed daily to CH_3_HgCl mixed in their diets at various concentrations. The phagocytic rate and phagocytic index demonstrated a significant decrease in all treatment groups compared with the control group from day 2 (*p* < 0.05; Figure 4). On days 14 and 28, the phagocytic rate of the 10 mg/kg group was significantly different between the treatment groups (*p* < 0.05). Moreover, the 10 mg/kg group had the lowest phagocytic rate and phagocytic index among the treatment groups during the study. In brief, the phagocytic rate and phagocytic index gradually decreased with increased concentration and more days of exposure to CH_3_HgCl.

**Figure 5:**
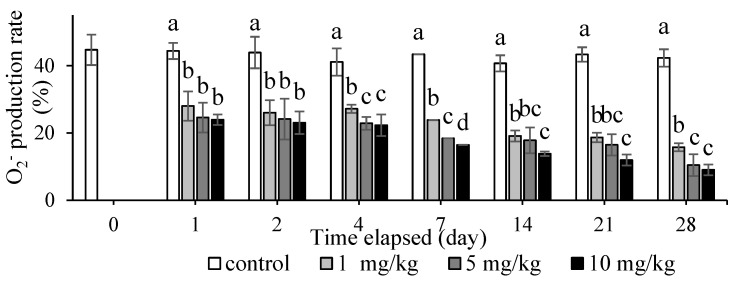
Superoxide anion production rate of hybrid grouper fed with various concentrations of methylmercury for 28 days. One-way ANOVA and Tukey’ test were performed to compare the differences between groups at indicated time point. Values are presented as mean ± SD (*n* = 3). Significant differences (*p* < 0.05) between treatment groups are indicated by different letters above the bars.

The O_2_^−^ production rate significantly decreased in all treatment groups compared with the control group from day 1 (*p* < 0.05; Figure 5). On days 1 and 2, no significant differences between the treatment groups were indicated. A significant difference between the treatment groups was observed on day 7 (*p* < 0.05); the 10 mg/kg group had the lowest O_2_^−^ production rate of the treatment groups. In brief, the O_2_^−^ production rate gradually decreased with increased concentration and more days of exposure to CH_3_HgCl.

### 3.4. Level of Methylmercury Bioaccumulation

The methylmercury content in the muscle tissue, liver and head kidney was analyzed using HPLC-ICP-MS (Table 1, Table 2, Table 3 and Table 4). For muscle tissue (Table 1), the accumulation of the 1, 5, and 10 mg kg ^−1^ treatment group was significantly higher (*p* < 0.05) on day 28 (224.60 ± 33.87, 427.50 ± 94.02, and 1213.17 ± 192.08 µg kg^−1^, respectively) compared with the accumulation on days 1, 4, 7, 14, and 21 (Table 1). On day 1, the 10 mg kg^−1^ treatment group was significantly different from the other treatment groups and the control group (*p* < 0.05). On day 4, the 5 and 10 mg kg^−1^ treatment groups significantly differed from the control group (*p* < 0.05). On day 7, a significant difference between the 10 mg kg^−1^ treatment group and the control group was indicated (*p* < 0.05). On day 14, significant differences between the 5 and 10 mg kg^−1^ treatment groups and the control group were indicated (*p* < 0.05). On days 21 and 28, all treatment groups were significantly different compared with the control group (*p* < 0.05). On day 28, significant differences between the three treatment groups were also indicated (*p* < 0.05).

Regarding the liver (Table 2), the bioaccumulation of the 1, 5, and 10 mg kg^−1^ treatment groups was significantly higher (*p* < 0.05) on day 28 (478.73 ± 51.69, 1136.67 ± 91.59, and 3428.00 ± 346.34 µg kg^−1^, respectively) compared with that on day 1, 4, 7, 14, and 21 (Table 2). On days 1 and 4, no significant difference between the treatment groups and the control group was indicated (*p* > 0.05). On day 7, a significant difference between the 10 mg kg^−1^ treatment group and the control group was indicated (*p* < 0.05). On days 14, 21, and 28, all of the treatment groups had significant differences compared with the control group (*p* < 0.05). Moreover, on day 28, significant differences between the three treatment groups were indicated (*p* < 0.05).

Regarding the head kidney (Table 3), the accumulation of the 1, 5, and 10 mg kg^−1^ treatment groups were significantly higher (*p* < 0.05) on day 28 (433.97 ± 14.48, 1063.67 ± 159.79, and 4037.67 ± 287.47 µg kg^−1^, respectively) compared with the accumulation on days 1, 4, 7, 14, and 21 (Table 3). On day 1, no significant difference between the treatment groups and the control group was indicated (*p* > 0.05). On days 4, 7, 14, and 21, all treatment groups had significant differences compared with the control group (*p* < 0.05). On day 28, all treatment groups had significant differences compared with the control group (*p* < 0.05); significant differences between the three treatment groups were also indicated (*p* < 0.05).

The accumulation of MeHg in the liver, head kidney, and muscle tissue gradually increased with increasing concentration and more days of exposure. Additionally, methylmercury exhibited significant accumulation on day 28 in all of the tissue samples of the treatment groups. The accumulation of MeHg in all the tissue samples was not significantly different at the start of the study. However, the accumulation increased gradually during the study and increased exponentially on day 28. At the conclusion of the study, the liver and head kidney accumulated more MeHg compared with the muscle tissue (Table 4).

## 4. Discussion

Organic mercury that is toxic to humans and animals is found in the forms of methylmercury and dimethylmercury. Methylmercury is a readily available form in nature that can accumulate in the food chain and be consumed by humans who eat fish [9]. Fish are usually exposed to MeHg through their feed (fish meal) and environmental water pollution [20]. The direct and indirect exposure of MeHg by fish can cause loss of appetite, brain damage, abnormal system development, cell necrosis, and the inhibition of respiratory burst activity, all of which affect the growth and immune response of fish and shellfish [21,22,23,24]. In this study, we evaluated data regarding the effect of MeHg exposure on nonspecific immune response and bioaccumulation in hybrid grouper.

Several studies have been conducted to examine MeHg toxicity on aquatic species cells in vitro. The SAF-1 cells from the marine gilthead seabream were immersed in various concentrations of MeHg and Hg for 24 h; the concentration for 50% of maximal effect (EC_50_) were 0.018 mM (≈4.519 ppm) and 0.3 mM (75.32 ppm), respectively [25]. Similarly, the HGST-BR green turtle brain cell line was exposed to 5 µM of MeHg and HgCl; cell viability in such exposure was 49% and 69%, respectively [26]. According to ISO 10993-5, a cell viability value that is <70% of the blank (control) has a cytotoxic potential to the host cell [27]. In our study, the head kidney leukocytes were incubated with various concentrations of CH_3_HgCl to determine the maximum concentration to use in the dietary exposure. The leukocytes are directly exposed to methylmercury in in vitro experiment, and the cells will be damaged. Therefore, we first perform cell viability test to confirm whether leukocytes are still viability after 30 min of exposure to different concentrations of methylmercury, especially at high concentrations. Figure 1 illustrates that the viability of cells treated with 1000, 5000, and 10,000 µg/L was 82%, 39% and 32%, respectively. Therefore, these results are in accordance with a relevant study that demonstrated that concentrations ˃ 1000 µg/L have a toxic effect on fish. We conducted further research to determine the effect of MeHg on bioaccumulation and nonspecific immune responses such as phagocytic activity and the O_2_^−^ production rate in hybrid grouper using concentrations of MeHg ˃ 1000 µg/L.

Phagocytic activity is a crucial immune response in organisms for resisting foreign pathogens. After phagocytic cells engulf foreign pathogens or apoptotic cells, they produce reactive oxygen species such as O_2_^−^ to remove the phagocytic substances [28,29]. In vitro approaches are used to observe the effects of MeHg exposure, particularly on nonspecific immune responses of aquatic species including phagocytic activity and O_2_^−^ production rate. A relevant study demonstrated that Mediterranean mussel (*Mytilus galloprovincialis*) hemocytes incubated with 10^−7^, 10^−6^, or 10^−5^ M of MeHg had phagocytic rates that decreased with the increase of MeHg concentration and resulted in cell death [24]. Moreover, European sea bass (*Dicentrarchus labrax*) head kidney leukocytes that were incubated in 0.0005, 0.005, 0.05, or 0.1 mM of MeHg had phagocytic rates and respiratory burst activity that were significantly lower than those of that study’s control group (*p* < 0.05) [22]. Similarly, in our study, the phagocytic activity and O_2_^−^ production rate of head kidney leukocytes decreased at concentrations ˃ 500 and 200 µg/L, respectively. The phagocytic activity and O_2_^−^ production rate decreased with the increase of MeHg concentrations. This is because the number of living cells is reduced, resulting in a reduced immune system response [27].

Hybrid groupers were exposed daily to CH_3_HgCl at various concentrations mixed in their diets to determine the nonspecific immune responses in the leukocytes of the head kidney. Head kidney leukocytes were used at several time points of sampling to evaluate the phagocytic activity and O_2_^−^ production rate (Figure 4 and Figure 5). The head kidney of fish plays a crucial role in the endocrine response to stress and the hematopoietic response [30]. This study demonstrated that MeHg exposure can reduce the phagocytic rate, phagocytic index, and O_2_^−^ production rate in head kidney leukocytes of the hybrid grouper. This finding is similar to that of a relevant study that found that the phagocytic activity of *Mya arenaria* clams hemocytes decreased after exposure to mercury chloride and methylmercury at a concentration of 10^6^ M in water for 28 days [31]. Nile tilapia (*Oreochromis niloticus*) exposed to mercury chloride at a concentration of 0.05 mg/kg for 21 days exhibited a decrease in the phagocytic index, phagocytic rate, and macrophage oxidative burst index compared with that study’s control group [32]. Large yellow croaker (*Pseudosciaena crocea*) immersed in methylmercury at concentrations of 0.25, 1, and 4 µg/L for 30 days was significantly decreased superoxide dismutase activity compared with the control group [23]. Moreover, the sampling days of this study were designed according to the characteristics of the immune response. The immune reactions can be divided into acute and chronic reactions according to the time when the immune response occurs. Acute immune reactions occur on day 1, 4, and 7 in the experiment. The chronic reactions occur on day 14, 21 and 28 in the experiment. This result indicated MeHg affects the immunity of hybrid grouper very quickly, whether the concentration of MeHg is high or low. Thus, environmental stressors including MeHg exposure can affect the physiological state and overall welfare of fish.

Because few studies have focused on MeHg toxicity due to its bioaccumulation in hybrid grouper tissues after oral exposure, we developed a feeding study to better understand the bioaccumulation of MeHg in the liver, head kidney, and muscle tissue. A relevant study observed more Hg accumulation in muscle tissue than in the gills, liver, and gonads of perch (*Perca fluviatilis*) [33]. Similarly, according to Järv et al. [34], the highest concentration of Hg in perch (*P. fluviatilis*) was found in muscle tissue rather than the liver. By contrast, our study demonstrated that MeHg more significantly accumulated in the liver and head kidney than it did in the muscle tissue. This result was similar to that of a relevant study in which white sturgeon (*Acipenser transmontanus*) injected with 250, 500, or 1000 µg/kg of methylmercury chloride exhibited significant Hg accumulation in the intestines, followed in significance by the head kidney, liver, and muscle tissue [35]. Similarly, a study of olive flounder (*Paralichthys olivaceu*) fed with diets containing MeHg concentrations of 0, 10, 20, 40, or 160 mg/kg for 8 weeks indicated that MeHg accumulated primarily in the head kidney, followed in significance by the liver and gill tissues [13]. These results are in accordance with the findings in our study. The bioaccumulation of heavy metal is related to detoxification mechanisms and metabolism [36]. In marine mammals’ livers, MeHg can be transformed into inorganic mercury, which is a less toxic form of mercury. When inorganic mercury is combined with metallothionein, the toxicity of mercury to organisms can be reduced [37]. However, high concentrations of mercury are still harmful to organisms. Pathological aberrations in the liver and head kidney tissues observed in histopathology of the Nile tilapia (*O. niloticus*) after 50 mg/kg Hg exposure have been discovered [38]. Bioaccumulation be an enormous increase between days 21 and 28 which would possibly suggest systems are overwhelmed and Hg cannot be eliminated. However, this experiment analyzed the biological hazards and accumulation of MeHg to fish under different doses and exposure times. The results indicate that hybrid grouper significantly accumulated MeHg in the liver and head kidney rather than the muscle tissue and higher concentrations and more exposure days resulted in more significant accumulation.

Taken together, cell viability in vitro indicated that exposure to a concentration of MeHg exceeding 1000 µg/L have a toxic effect on cells, which decreases cell viability. This result correlates with the decreasing immune response at higher concentrations of MeHg exposure, which reduces the biological function of phagocytosis, causes cell death, and ultimately leads to a decline in fish immunity. The detoxification and metabolism activity in the liver and head kidney may become disturbed, and this disturbance may result in higher bioaccumulation of MeHg.

## 5. Conclusions

The initial study was evaluated the methylmercury exposure of hybrid grouper head kidney leukocytes in vitro subjected to different concentrations of MeHg. By treating head kidney leukocytes in MeHg solution, we found that MeHg is an pollutant that causes a decrease in cell viability and nonspecific immune response such as phagocytic rate and superoxide anion production rate. During foodborne of exposure, we have also demonstrated that MeHg exposure can decrease the phagocytic rate, phagocytic index, and superoxide anion production rate. After dietary exposure to MeHg for 28 days, hybrid grouper significantly accumulated MeHg in the liver and head kidney rather than the muscle tissue. The cumulative concentration of each organ was positively correlated with the experimental concentration and exposure time. Therefore, this result correlates with the decreasing immune response at higher concentrations of MeHg exposure, which reduces the biological function and may relate to the liver and head kidney’s detoxification and metabolism mechanism resulting in the increased accumulation of MeHg.

## Figures and Tables

**Table 1:** Bioaccumulation of methylmercury (µg/kg) in muscle tissue of hybrid grouper fed with various concentrations of methylmercury for 28 days.

Dose (mg/kg)	Time Elapsed (Day)						
	0	1	4	7	14	21	28
Control	40.56 ± 3.10 ^B^	33.73 ± 2.75 _b_^CD^	25.40 ± 1.07 _b_^D^	47.37 ± 3.09 _b_^A^	33.17 ± 1.89 _c_^CD^	28.37 ± 0.05 _c_^D^	39.83 ± 0.83 _d_^BC^
1 mg/kg		38.57 ± 2.25 _b_^B^	31.90 ± 3.09 _ab_^B^	76.57 ± 9.90 _ab_^B^	71.40 ± 6.14 _b_^B^	71.50 ± 6.33 _b_^B^	224.60 ± 33.87 _c_^A^
5 mg/kg		33.13 ± 1.76 _b_^B^	50.00 ± 5.83 _a_^B^	89.17 ± 6.97 _ab_^B^	116.53 ± 2.91 _a_^B^	154.60 ± 32.74 _ab_^B^	427.50 ± 94.02 _b_^A^
10 mg/kg		42.07 ± 2.46 _a_^B^	46.27 ± 9.71 _a_^B^	130.27 ± 34.72 _a_^B^	130.07 ± 14.08 _a_^B^	177.80 ± 72.07 _a_^B^	1213.17 ± 192.08 _a_^A^

Values are shown as the mean ± SD (*n* = 3). One-way ANOVA and Tukey’ test were performed to compare the differences between groups at indicated time point or between different time points in a group.; Means in the same row with different letters (^A, B, C, D^) are significantly different (*p* < 0.05); Means in the same column with different letters (_a, b, c, d_) are significantly different (*p* < 0.05).

**Table 2:** Bioaccumulation of methylmercury (µg/kg) in liver of hybrid grouper fed with various concentrations of methylmercury for 28 days.

Dose (mg/kg)	Time Elapsed (Day)						
	0	1	4	7	14	21	28
Control	19.4 ± 0.75 ^B^	28.00 ± 1.02 _a_^B^	24.43 ± 5.83 _a_^B^	34.23 ± 0.68 _b_^A^	18.77 ± 4.46 _c_^B^	26.13 ± 2.19 _c_^B^	29.57 ± 1.23 _d_^B^
1 mg/kg		33.30 ± 1.40 _a_^C^	31.63 ± 9.97 _a_^C^	133.13 ± 32.26 _ab_^B^	159.63 ± 18.35 _b_^B^	218.57 ± 11.48 _b_^B^	478.73 ± 51.69 _c_^A^
5 mg/kg		30.23 ± 1.52 _a_^C^	53.83 ± 26.44 _a_^C^	134.63 ± 16.41 _ab_^C^	408.00 ± 43.29 _a_^B^	374.63 ± 76.46 _ab_^B^	1136.67 ± 91.59 _b_^A^
10 mg/kg		51.63 ± 20.74 _a_^B^	93.53 ± 38.30 _a_^B^	249.50 ± 97.37 _a_^B^	458.77 ± 90.92 _a_^B^	331.67 ± 44.73 _a_^B^	3428.0 ± 346.34 _a_^A^

Values are shown as the mean ± SD (*n* = 3). One-way ANOVA and Tukey’ test were performed to compare the differences between groups at indicated time point or between different time points in a group. Means in the same row with different letters (^A, B, C^) are significantly different (*p* < 0.05); Means in the same column with different letters (_a, b, c, d_) are significantly different (*p* < 0.05).

**Table 3:** Bioaccumulation of methylmercury (µg/kg) in head kidney of hybrid grouper fed with various concentrations of methylmercury for 28 days.

Dose (mg/kg)	Time Elapsed (Day)						
	0	1	4	7	14	21	28
Control	18.77 ± 3.51 ^A^	10.83 ± 0.54 _a_^B^	6.17 ± 1.05 _c_^B^	22.93 ± 0.40 _b_^A^	5.43 ± 1.25 _c_^B^	10.60 ± 0.28 _c_^B^	17.87 ± 1.51 _d_^A^
1 mg/kg		13.23 ± 2.41 _a_^C^	23.87 ± 6.33 _b_^C^	128.77 ± 56.34 _a_^B^	149.33 ± 19.01 _b_^B^	211.63 ± 16.16 _b_^B^	433.97 ± 14.48 _c_^A^
5 mg/kg		21.10 ± 1.61 _a_^D^	56.93 ± 13.70 _a_^CD^	189.13 ± 35.01 _a_^BCD^	266.00 ± 29.12 _a_^BC^	336.57 ± 50.95 _a_^B^	1063.67 ± 159.79 _b_^A^
10 mg/kg		36.30 ± 19.04 _a_^B^	103.87 ± 59.37 _a_^B^	222.70 ± 79.69 _a_^B^	316.73 ± 53.88 _a_^B^	252.93 ± 75.78 _a_^B^	4037.67 ± 287.47 _a_^A^

One-way ANOVA and Tukey’ test were performed to compare the differences between groups. Values are shown as the mean ± SD (*n* = 3); Means in the same row with different letters (^A, B, C, D^) are significantly different (*p* < 0.05); Means in the same column with different letters (_a, b, c, d_) are significantly different (*p* < 0.05).

**Table 4:** Bioaccumulation of methylmercury (µg/kg) in muscles, liver and kidney of hybrid grouper fed with various concentrations of methylmercury for 28 days.

Tissue	Dose (mg/kg)	Time Elapsed (Day)						
		0	1	4	7	14	21	28
Muscle	Control	40.56 ± 3.10 ^a^	33.73 ± 2.75 ^abc^	25.40 ± 1.07 ^ab^	47.37 ± 3.09 ^cd^	33.17 ± 1.89 ^ef^	28.37 ± 0.05 ^fg^	39.83 ± 0.83 ^d^
	1 mg/kg		38.57 ± 2.25 ^abc^	31.90 ± 3.09 ^ab^	76.57 ± 9.90 ^bcd^	71.40 ± 6.14 ^ef^	71.50 ± 6.33 ^efg^	224.60 ± 33.87 ^d^
	5 mg/kg		33.13 ± 1.76 ^abc^	50.00 ± 5.83 ^ab^	89.17 ± 6.97 ^bcd^	116.53 ± 2.91 ^ef^	154.60 ± 32.74 ^defg^	427.50 ± 94.02 ^d^
	10 mg/kg		42.07 ± 2.46 ^ab^	46.27 ± 9.71 ^ab^	130.27 ± 34.72 ^abcd^	130.07 ± 14.08 ^ef^	177.80 ± 72.07 ^cdef^	1213.17 ± 192.08 ^c^
Liver	Control	19.4 ± 0.75^b^	28.00 ± 1.02 ^abc^	24.43 ± 5.83 ^ab^	34.23 ± 0.68 ^cd^	18.77 ± 4.46 ^f^	26.13 ± 2.19 ^fg^	29.57 ± 1.23 ^d^
	1 mg/kg		33.30 ± 1.40 ^abc^	31.63 ± 9.97 ^ab^	133.13 ± 32.26 ^abcd^	159.63 ± 18.35 ^de^	218.57 ± 11.48 ^abcde^	478.73 ± 51.69 ^d^
	5 mg/kg		30.23 ± 1.52 ^abc^	53.83 ± 26.44 ^ab^	134.63 ± 16.41 ^abcd^	408.00 ± 43.29 ^ab^	374.63 ± 76.46 ^a^	1136.67 ± 91.59 ^c^
	10 mg/kg		51.63 ± 20.74 ^a^	93.53 ± 38.30 ^a^	249.50 ± 97.37 ^a^	458.77 ± 90.92 ^a^	331.67 ± 44.73 ^abc^	3428.00 ± 346.34 ^b^
Kidney	Control	18.77 ± 3.51^b^	10.83 ± 0.54 ^c^	6.17 ± 1.05 ^b^	22.93 ± 0.40 ^d^	5.43 ± 1.25 ^f^	10.60 ± 0.28 ^g^	17.87 ± 1.51 ^d^
	1 mg/kg		13.23 ± 2.41 ^bc^	23.87 ± 6.33 ^ab^	128.77 ± 56.34 ^abcd^	149.33 ± 19.01 ^de^	211.63 ± 16.16 ^bcde^	433.97 ± 14.48 ^d^
	5 mg/kg		21.10 ± 1.61 ^bc^	56.93 ± 13.70 ^ab^	189.13 ± 35.01 ^abcd^	266.00 ± 29.12 ^cd^	336.57 ± 50.95 ^ab^	1063.67 ± 159.79 ^c^
	10 mg/kg		36.30 ± 19.04 ^abc^	103.87 ± 59.37 ^a^	222.70 ± 79.69 ^ab^	316.73 ± 53.88 ^bc^	252.93 ± 75.78 ^abcd^	4037.67 ± 287.47 ^a^

Two-way ANOVA and Tukey’ test were performed to compare the differences between groups and days of exposure. Values are shown as the mean ± SD (*n* = 3); Means in the same column with different letters (^a–g^) are significantly different (*p* < 0.05).

## Data Availability

The authors confirm that the data supporting the findings of this study are available within the article.
